# Whole-genome sequencing of adenovirus 41 directly from wastewater using nested overlapping PCR and MinION

**DOI:** 10.1128/spectrum.00471-26

**Published:** 2026-08-05

**Authors:** Naziba Nusrat, John S. Meschke, Rachel Swanstrom, Roberto A. Rodríguez

**Affiliations:** 1 Department of Environmental and Occupational Health Sciences, Bloomington-School of Public Health, Indiana University 41473, Bloomington, Indiana, USA; 2 Environmental and Occupational Health Sciences, School of Public Health, University of Washington 167466, Seattle, Washington, USA; Universidad Tecnica de Ambato, Ambato, Ecuador

**Keywords:** enteric adenovirus, whole-genome sequencing, wastewater-based epidemiology

## Abstract

Human adenovirus F41 (HAdV-F41) is one of the leading causes of children’s acute gastroenteritis and was recently linked to an outbreak of severe acute hepatitis of unknown etiology among children during 2021 to 2022. While most evidence is based on clinical data, wastewater-based epidemiology offers a community-level approach to monitoring circulating strains and enhancing outbreak preparedness. In this study, we developed an overlapping amplicon-based whole-genome sequencing approach to directly detect HAdV-F41 from archived wastewater samples, using nested PCR with 13 primer sets. Archived wastewater samples were collected between 2021 and 2022 from three treatment plants in Seattle, USA. The viral load ranged from 1.2 × 10^3^ to 8.4 × 10^3^ genome copies per liter. The Oxford Nanopore platform was used for whole-genome sequencing. Complete or partial (>84%) HAdV-F41 genomes were recovered from wastewater samples, with mean coverage depths ranging from 10³ to 10⁵. The consensus sequences showed more than 99% similarity to reference genomes in the NCBI database. The phylogenetic analysis revealed that 2 sequences clustered within lineage 2a and 11 within lineage 2b, reflecting that at least two sub-lineages were circulating in the community at that time. Our results demonstrate that the overlapping amplicon-based whole-genome sequencing approach using the Oxford Nanopore platform reliably recovers HAdV-F41 genomes from wastewater. This method offers high-resolution genomic surveillance of circulating, clinically relevant HAdV-F41, supporting wastewater-based epidemiology as a valuable tool for detecting emerging variants and strengthening the early warning system for future disease outbreaks.

**IMPORTANCE:**

Human adenovirus F41 is a primary cause of childhood gastroenteritis and has been linked to recent outbreaks of severe acute hepatitis in children, yet community-level genomic surveillance of this virus remains limited. This study shows that wastewater can be used to recover nearly complete HAdV-F41 genomes through a targeted overlapping-amplicon sequencing strategy on the Oxford Nanopore platform. By applying this method to archived wastewater samples, we detected the simultaneous circulation of multiple viral lineages in a large city. These findings extend wastewater-based epidemiology beyond SARS-CoV-2 and emphasize its importance for monitoring clinically significant enteric viruses. The method described here offers a scalable tool for tracking viral evolution in communities and enhancing early warning systems for future outbreaks.

## INTRODUCTION

Adenovirus is one of the common microbial water contaminants with potential threats to public health (1). Human adenoviruses (HAdVs) are associated with a vast spectrum of diseases, depending on their tissue tropism, ranging from respiratory illnesses to gastroenteritis (2–4). HAdVs are non-enveloped, double-stranded DNA viruses with an icosahedral shape, classified under the genus *Mastadenovirus* (5). The genome of human adenoviruses spans about ∼35 kbp, capable of producing about 30–40 proteins (6). HAdVs are classified into seven categories (A–G) according to the percentages of guanine and cytosine in their genome and other biochemical and phylogenetic standards, which are subdivided into more than 100 subspecies (6–8).

The severity of HAdV infection varies according to age, immunity, and health condition (8). Enteric HAdVs, including adenoviruses from groups F (types 40 and 41) and G (type 52), are mainly associated with gastroenteritis (8–10). Specifically, HAdV-F41 is the second leading cause of acute gastroenteritis among children after rotavirus, responsible for 2.8% to 11.8% of infant diarrheal cases (11). According to previous studies, HAdV type F40/41 has been evidenced to develop most cases of childhood gastroenteritis among all the types and subtypes of HAdVs (8, 10). Its significant role in pediatric health arises from its capability of persisting in the gastrointestinal tract, transmitting through the fecal-oral route, and leading to widespread contamination of the water system (8, 11).

The structural and functional properties of HAdV-F determine its ability to infect host cells and the severity of the disease (12). The capsid protein structure of HAdVs consists of 240 hexons, 12 penton bases, and 12 fibers (13). Unlike other adenoviruses, HAdV-F40/41 possesses two kinds of fiber proteins: the long fiber (LF) binds to the cellular coxsackie-adenovirus receptor, and the short fiber (SF) binds to the heparan sulfate receptor (14–17). Despite sharing genetic similarities, HAdV-F41 has evolved to exhibit a more pronounced clinical impact than HAdV-F40 (12). HAdV-F41 has a unique arrangement and distinct surface charge distribution of capsid protein IX, which distinguishes it from other adenoviruses (12). A phylogenetic study published in 2022 about 32 HAdV-F41 genomes revealed that three lineages of HAdV-F41 co-circulate together: lineage 1, which includes the 1973 prototype “Tak” along with six isolates from 2007 to 2017; lineage 2, consisting of isolates from 2000 to 2022; and lineage 3, comprising isolates detected between 2009 and 2022 (6). Lineages 2 and 3 were subdivided into two sub-lineages (6). Lineage 3 is characterized by 45-nucleotide deletions in the long-fiber gene and notable genetic alterations in the short-fiber and E3 regions (6). Four genes encoding capsid proteins (hexon, penton, short-fiber, and long-fiber) are responsible for host cell binding during infection, which contain hypervariable regions (18). The genomic evolution of HAdV-F41 over the past decade may have influenced its virulence and cell tropism, leading to the emergence of novel variants with distinct disease characteristics, underscoring the need for extensive genomic studies on circulating HAdVs (2, 6, 19).

Epidemiological studies across various geographic regions have investigated the prevalence of HAdV-F41 in individuals presenting clinical symptoms. A high burden of diarrheal diseases among young children has been evidenced in low- and middle-income countries due to enteric HAdVs (20). A case-control study was conducted in seven low- and middle-income countries (Bangladesh, India, Pakistan, The Gambia, Kenya, Mali, and Mozambique) to measure the population-based burden and microbiological etiology of moderate to severe diarrhea among children under 5 years old (21). Although the study found the prevalence of enteric HAdV-F40/41 to be relatively low compared to other enteric pathogens (21), a subsequent reanalysis of the stool samples revealed a fivefold increase in the prevalence of enteric HAdV-F40/41, establishing it as one of the most common enteric pathogens significantly associated with child diarrhea (22). Beyond its extensively reported symptoms of gastroenteritis, recent articles have demonstrated a potential association between HAdV-F, particularly HAdV-F41, and the outbreak of severe acute hepatitis of unknown etiology (AHUE) among young children during late 2021 and early 2022 (23–26). AHUE was first reported in the United Kingdom in March 2021, and similar cases were eventually detected in various geographical locations, including the USA, Europe, Central America, and the Cluster of Asia, up to 2022 (27, 28). The WHO reported 1,010 probable cases of AHUE worldwide as of 8 July 2022 (29). Between 1 January 2022 and 4 July 2022, a total of 274 cases of acute hepatitis were detected in the United Kingdom, of which 58 cases of children aged 1 to 10 years underwent successful genotyping that revealed an association of HAdV-F41 with hepatitis (30). In the USA, the first case was reported in Alabama in October 2021, and as of May 2022, a total of 107 cases were recorded (24, 28). These cases highlight the potential of HAdV-F41 to cause significant outbreaks, potentially leading to the global dissemination of circulating viruses.

Most epidemiological studies on HAdVs focus on detecting viral circulation in human specimens, such as respiratory samples, stool, blood, ocular swabs, serum, urine, and tissue, primarily from individuals with clinical symptoms (22, 31–33). Most current evidence of HAdV-F41 circulation relies on detection of viruses in stool specimens from children with acute gastroenteritis (32, 34–38). However, asymptomatic and silent virus transmission remains undetectable by clinical methods. The concept of wastewater-based epidemiology (WBE) offers a valuable opportunity to detect viruses at the population level, identify infection hotspots, and serve as an early warning system for emerging outbreaks (39, 40). This method enhances an opportunity for timely public health interventions and augments the benefit of traditional epidemiological surveillance methods (40). Wastewater surveillance for virus detection has previously been utilized in poliovirus monitoring (41, 42). With the emergence of COVID-19, WBE has further highlighted its crucial role in viral detection (43, 44). Studies have demonstrated the presence of HAdV-F40/41 in wastewater, supporting the potential of WBE as an indicator of community-wide transmission of enteric viral species (19, 26). Research has shown that the prevalence of HAdV-F correlates with the clinical specimen positivity rate, which indicates the effectiveness of WBE as a surveillance tool for HAdV-F shedding and silent transmission in the community (19, 26, 45). Additionally, wastewater surveillance has also provided evidence linking HAdV-F to cases of severe acute hepatitis (19, 26).

Despite some data existing on the prevalence of HAdV-F41 in WBE (26), the retrieval of complete genome sequences from viruses circulating in wastewater remains a significant challenge. While specific studies have reported molecular detection of HAdV-F41, they primarily focus on specific viral genes rather than whole-genome sequencing (19), leaving a significant gap in our understanding of the complete genomic map (pangenome) of HAdV-F41 in wastewater. Maes et al. (2) followed an overlapping amplicon-based sequencing approach with Oxford Nanopore MinION technology to detect circulating F41 viral lineages from East England (2). This approach is advantageous because it allows for the complete genomic data of the virus to be obtained without the need for virus growth on cell lines (2). This paper describes the development and application of a nested, overlapping amplicon-based method for sequencing the complete adenovirus 41 genome directly from wastewater, using samples collected for WBE.

## MATERIALS AND METHODS

### Sample collection and DNA extraction

Wastewater samples were collected from three wastewater treatment plants in Seattle, Washington, between 2021 and 2022. The samples were concentrated using PEG precipitation and then frozen by the Environmental & Occupational Health Microbiology Lab (EOHML) at the University of Washington. The PEG concentration was performed by adding PEG and NaCl to obtain a final concentration of 9% PEG and 0.3 M NaCl. The samples were incubated overnight, and then centrifuged at 4,200 × *g* for 1 hour. The pellet was resuspended in phosphate-buffered saline (1×, pH 7.2). The initial volume of collected environmental samples was 200 mL, which was concentrated to 10 mL. Nucleic acid extraction of concentrated samples was performed using the QIAamp MinElute Virus Spin Kit, following the manufacturer’s protocol. A total sample volume of 200 µL was used for each extraction, and nucleic acids were eluted with 150 µL of elution buffer. Samples were screened for human adenovirus F40/41 positivity using a dPCR assay targeting the fiber gene region, as described by reference 46. The QIAcuity Digital PCR System was used to quantify gene targets. Following the protocol of QIAcuity Probe PCR Master Mix, each reaction mix was prepared with 10 µL of 4× QIAcuity Probe PCR Master Mix, 4 µL of 10× primer/probe mixture (0.8 µM forward primer, 0.8 µM reverse primer, and 0.4 µM probe as the final concentration), 5 µL of template DNA (sample), and 21 µL of nuclease-free water to reach a volume of 40 µL. The reaction mixtures were vortexed and centrifuged, then transferred to a 24-well QIAcuity 26k nanoplate. The nanoplate was transferred to the QIAcuity Digital PCR instrument, and the targeted gene from each sample was amplified to estimate the concentration of virus in gene copies (GC) by detecting the signal from the fluorescence probe (FAM). The two-step thermal cycling was carried out for amplification using the following conditions: (i) initial denaturation at 95°C for 3 minutes; (ii) 45 cycles of amplification consisting of (a) denaturation at 95°C for 5 seconds and (b) annealing and extension at 60°C for 1 minute and 30 seconds.

For analysis, units were converted to GC/L using the following equation, which was prepared based on the information provided by EOHML at the University of Washington for preparing concentrated samples and the sample volume used for extraction and elution:


$$Virus\frac{GC}{L}=\frac{\left(\frac{GC}{µL}\right)per\;reaction\times Vr\times Ve\times Vc}{Vt\times Vs\times Vi}\times 1000$$

where $Virus\frac{GC}{L}$ = virus concentration in raw sample from the environment; $\left(\frac{GC}{µL}\right)per\;reaction$ = value obtained from dPCR result; Vr = final dPCR reaction volume (40 µL); Ve = elution vol (150 µL); Vc = concentration vol of sample after PEG concentration (10 mL); Vt = volume of DNA template used for PCR reaction (5 µL); Vs = volume of concentrated sample used for nuclear extraction (200 µL); Vi = initial volume of sample (200 mL).

The positive samples were taken for further analysis, including whole-genome sequencing.

### Primer design for whole-genome sequencing with overlapping regions

Whole-genome sequences of 111 human adenovirus F41 isolates, approximately 34 to 35 kbp in length, were obtained from GenBank in FASTA format and used for primer design (Table S1). Sequences were aligned using ClustalW in the MEGA X statistical package. The conserved regions showing 100% identity across the entire primer length in all 111 genomes were targeted for primer selection. For primer design, we targeted a melting temperature (Tm) range of 55°C–60°C. A nested PCR approach was employed to ensure amplification specificity. Thirteen overlapping outer primer sets for the first-step PCR reaction, and 13 nested primer sets for the corresponding nested PCR reactions were designed to amplify approximately ~4,000–6,500 bp tiled amplicons across the entire genome (Table S2). Overlaps of approximately 2,500 bp were maintained between adjacent amplicons.

### PCR amplification of viral genomes

All the adenovirus-positive samples were subjected to further analysis. The targeted amplicons for each sample were produced following a nested PCR approach. PCR amplification was performed using 2× Invitrogen Platinum SuperFi II PCR Master Mix with a final primer concentration of 0.5 µM and 5 µL of sample per 20 µL reaction to generate the first-step PCR product. Two microliters of the first-step PCR products was used to create nested PCR products, maintaining a similar concentration of primers and master mix. A three-step thermal cycling protocol was employed as follows: (i) initial denaturation at 98°C for 30 seconds, (ii) 35 cycles of amplification consisting of (a) denaturation at 98°C for 10 seconds; (b) annealing at 60°C for 10 seconds; (c) extension at 72°C for 5 minutes; (iii) final extension at 72°C for 5 minutes.

Following nested amplification, the final PCR products were corroborated by gel electrophoresis with 1% agarose stained with SYBR Safe to confirm the presence and size of the amplified amplicons. A 1 kb DNA ladder was used to estimate the size of the amplified amplicons. The presence of distinct bands corresponding to ~4,000–6,500 bp was confirmed by visualization of the gel (Fig. 2; Fig. S1).

For the wastewater samples, the resulting amplicons were subsequently pooled by combining 10 µL from each raw amplicon, creating composite samples for purification. Each pooled sample was purified using the QIAamp DNA Mini Kit (Qiagen) according to the manufacturer’s instructions.

### Nanopore sequencing and data processing

Sequencing libraries were prepared using the Oxford Nanopore Technologies Native Barcoding Kit 24 V14 (SQK-NBD114.24) following the standard protocol. Library preparation was carried out using approximately 200 fmol (650 ng) of pooled DNA, which underwent sequential steps of end-repair, barcoding ligation, and adapter ligation. Samples were loaded onto an R10.4.1 flow cell, and the sequencing was initiated using MinKNOW software. Raw data acquisition and basecalling were performed using the MinKNOW software with the Eldorado application, using the high-accuracy model (dna_r10.4.1_e8.2_400bps_hac@v4.3.0). The basecalled sequencing data (FASTQ) were further analyzed using the Galaxy server (https://usegalaxy.org). The resulting FASTQ reads were filtered to reads 4,100–6,000 base pairs and assembled *de novo* with Flye (version 2.9.6). The resulting contigs served as the basis for generating consensus sequences. To improve accuracy, the sequences were polished using the Medaka consensus pipeline (version 1.7.2), a tool that utilizes a neural network for assembly polishing. The model selected for Medaka consensus calling was r1041_e82_400bps_hac_g615. For the majority of samples, *de novo* assembly using Flye generated a single contig spanning the complete or near-complete genome. These assemblies were subsequently polished using long-read data and then queried against reference databases using Basic Local Alignment Search Tool (BLAST) to identify the closest related sequences.

For samples in which assembly resulted in multiple contigs collectively covering the genome, each contig was first subjected to BLAST analysis to assess sequence identity and orientation; orientations were corrected as necessary. Contigs were then aligned to a reference genome using Minimap2 to determine their relative positions and to identify overlapping regions. Where overlaps were detected, contigs were merged to generate a scaffolded assembly; regions lacking overlap were retained as gaps.

Gap regions and contig junctions were further refined through additional polishing with Medaka, conditional on read support. The resulting assemblies were subsequently re-evaluated by BLAST to identify the most closely related reference sequences, which were used to estimate genome completeness and support downstream comparative analyses.

The final consensus sequences served as the reference for subsequent distribution of sequencing depth coverage across the genome. Samtools depth (version 1.15.1) was used to calculate the per-base read depth of each sequence. The mean depths were calculated using the following equation, as shown in Table 1:


$$Mean\;Depth=\frac{Total\;number\;of\;bases\;in\;all\;mapped\;reads}{Length\;of\;sequences\;in\;base\;pairs}$$

**TABLE 1:** Overview of samples, sequencing data, genomic assemblies, and BLAST search outcomes^*a*^

Sample site and date of collection	ID	Reads N50	Assembly length	Mean read depth	BLAST result							
					Description	Location	Query cover	E value	Per. Ident.	Acc. Len	Accession	% of Assembly completeness (based on BLAST accessions)
South plant retreatment plant, 12 September 2022	BC 1	4,912	34,140	4,966.16	Human mastadenovirus F Kobe180227 DNA, complete genome	Japan	100%	0	99.96	34,170	LC791124.1	99.91
South plant retreatment plant, 9 August 2022	BC 2	4,967	34,105	8,823.52	Human adenovirus 41 isolate HOUA-GOSH08, complete genome	United Kingdom	100%	0	99.97	34,166	OP174917.1	99.82
West Point treatment plant, 11 July 2022	BC 3	4,875	34,110	5,018.62	Human adenovirus 41 isolate HOUA-GOSH08, complete genome	United Kingdom	100%	0	99.98	34,166	OP174917.1	99.84
Brightwater treatment plant, 1 November 2022	BC 4	4,926	34,122	7,857.36	Human adenovirus 41 isolate Hannover_20217_2, complete genome	Germany	100%	0	99.96	34,185	ON532821.1	99.82
West Point treatment plant, 1 November 2021	BC 5	4,961	34,144	11,702.97	Human mastadenovirus F Kobe180227 DNA, complete genome	Japan	100%	0	99.98	34,170	LC791124.1	99.92
South plant retreatment plant, 7 December 2021	BC 7	4,996	34,102	7,885.55	Human mastadenovirus F Kobe180227 DNA, complete genome	Japan	100%	0	99.96	34,170	LC791124.1	99.80
Brightwater treatment plant, 1 November 2021	BC 8	4,933	34,122	6,830.52	Human adenovirus 41, complete genome	Spain	100%	0	99.95	34,175	ON561778.1	99.84
Brightwater treatment plant, 6 December 2021	BC 9	4921	34,120	3,310.40	Human adenovirus 41 isolate HOUA-GOSH08, complete genome	United Kingdom	100%	0	99.88	34,166	OP174917.1	99.87
Brightwater treatment plant, 12 September 2022	BC 10	4,898	34,110	9,745.09	Human mastadenovirus F Kobe180227 DNA, complete genome	Japan	100%	0	99.99	34,170	LC791124.1	99.82
West Point treatment plant, 1 August 2022	BC 11	4,960	34,122	8,691.51	Human adenovirus 41 isolate MU22, complete genome	Iraq	100%	0	99.91	34,172	MG925782.1	99.85
West Point treatment plant, 13 June 2022	BC 12	4,928	29,004	5,093.68	Human adenovirus 41 isolate HOUA-GOSH08, complete genome	United Kingdom	100%	0	99.99	34,166	OP174917.1	84.89
Brightwater treatment plant, 11 July 2022	BC 13	4,881	34,165	8,316.87	Human adenovirus 41 isolate Hannover_20217_2, complete genome	Germany	100%	0	99.84	34,185	ON532821.1	99.94
West Point treatment plant, 12 September 2022	BC 14	4,867	33,289	10,083.33	Human adenovirus 41 isolate MU22, complete genome	Iraq	100%	0	99.98	34,172	MG925782.1	97.42
BC 15 tak reference (positive control)	BC 15	4,923	32,023	10,058.13	Human adenovirus 41 isolate Tak, complete genome	Netherlands	100%	0	99.99%	34,188	DQ315364.2	93.67

^*a*^ Reads N50 represents the median read length at which 50% of the total sequencing bases are contained in reads of equal or greater length. Assembly length corresponds to the total length (bp) of the final consensus genome assembly generated for each sample. Mean read depth indicates the average sequencing coverage across the assembled genome. BLAST results include the closest reference genome identified in the NCBI nucleotide database using BLASTn, along with query coverage, E-value, percent identity, accession length, and accession number. Percent assembly completeness was calculated as the ratio of the assembled genome length to the length of the closest BLAST reference accession. “Per. Ident.” = percent identity; “Acc. Len” = accession length.

For visualization, genome-wide depths were transformed to a log scale and plotted using RStudio (Fig. 3). This enabled the evaluation of depth distribution across the genome, highlighting amplification efficiency. The consensus sequences were searched using the BLAST, and information on closely related nucleotide accessions was retrieved. The closest related nucleotide accessions were selected based on the percentage of identity, E-value, and query coverage. The number of primary reads mapped to the accessions was measured using Samtools Flagstat on the Galaxy server. The information about the lengths of the accessions was used for the estimation of genome completeness following the equation:


$$\%\;Genome\;completeness=\frac{\left(Length\;of\;assembled\;genome\right)\times 100}{\left(Length\;of\;accession\right)}$$

Final sequences were used for downstream analysis, including phylogenetic analysis and lineage detection. The study sequences and human adenovirus F41 sequences retrieved from NCBI GenBank (Table S1) were aligned using MAFFT to generate multiple sequence alignments. A maximum likelihood phylogenetic tree was constructed incorporating both the study sequences and human adenovirus F41 sequences retrieved from GenBank using IQ-TREE (version 2.4.0). To provide statistical support for the branching pattern and enhance confidence in phylogenetic resolution, 1,000 ultrafast bootstrap replicates were conducted. Sequences were assigned to lineages based on clustering within the human adenovirus F41 lineage classification published by Götting et al. (6). In addition to whole-genome analysis, separate gene-level alignments and tree constructions for four genes (hexon, penton, long fiber, and short fiber) were conducted using a similar MAFFT and IQ-TREE workflow to investigate gene-specific evolutionary patterns and relationships among isolates across and within identified lineages. Annotation of all the phylogenetic trees was performed using iTOL v.7.

## RESULTS

**Fig 1:**
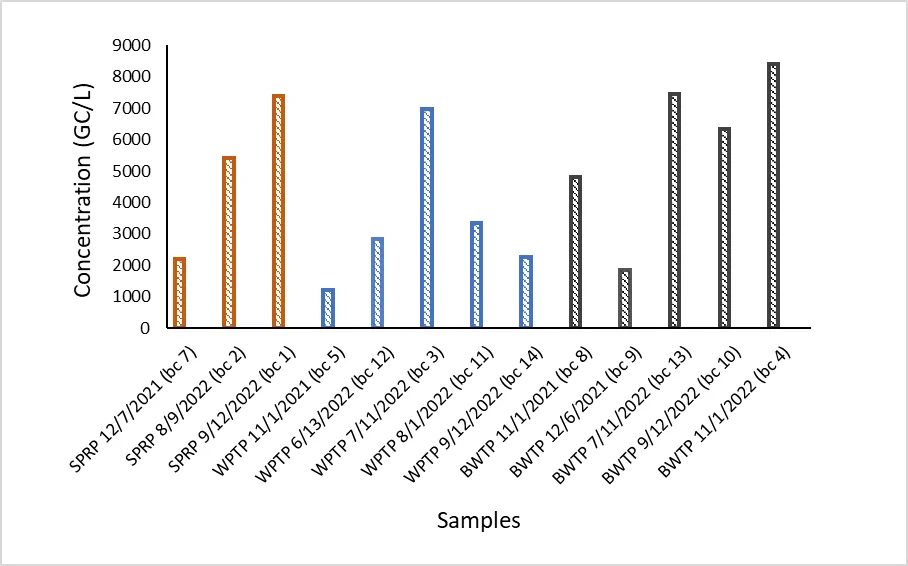
Adenovirus concentration measured in samples collected on different dates from multiple wastewater treatment plants. SPRP, South Plant Re-treatment Plant; WPTP, West Plant Re-treatment Plant; BWTP, Bright Water Treatment Plant.

All 13 archived wastewater samples collected from three treatment plants in Seattle, Washington, between 2021 and 2022, were positive for human adenovirus F41 using a digital PCR assay targeting the fiber gene region. Concentrations of human adenovirus F ranged from 1.2 × 10^3^ to 8.4 × 10^3^ GC/L, reflecting varying viral loads of the target genome across environmental samples, with variations of up to a 1 log scale (Fig. 1). During November 2021, the viral load was lowest (1.2 × 10^3^ GC/L) at the West Point treatment plant, and in 2022 it increased, peaking from July to September at all sites (6.9 × 10^3^ to 7.4 × 10^3^ GC/L) (Fig. 1). After September, data from the Brightwater treatment plant were only available, which rose again to 8.4 × 10^3^ GC/L in November 2022 (Fig. 1).

**Fig 2:**
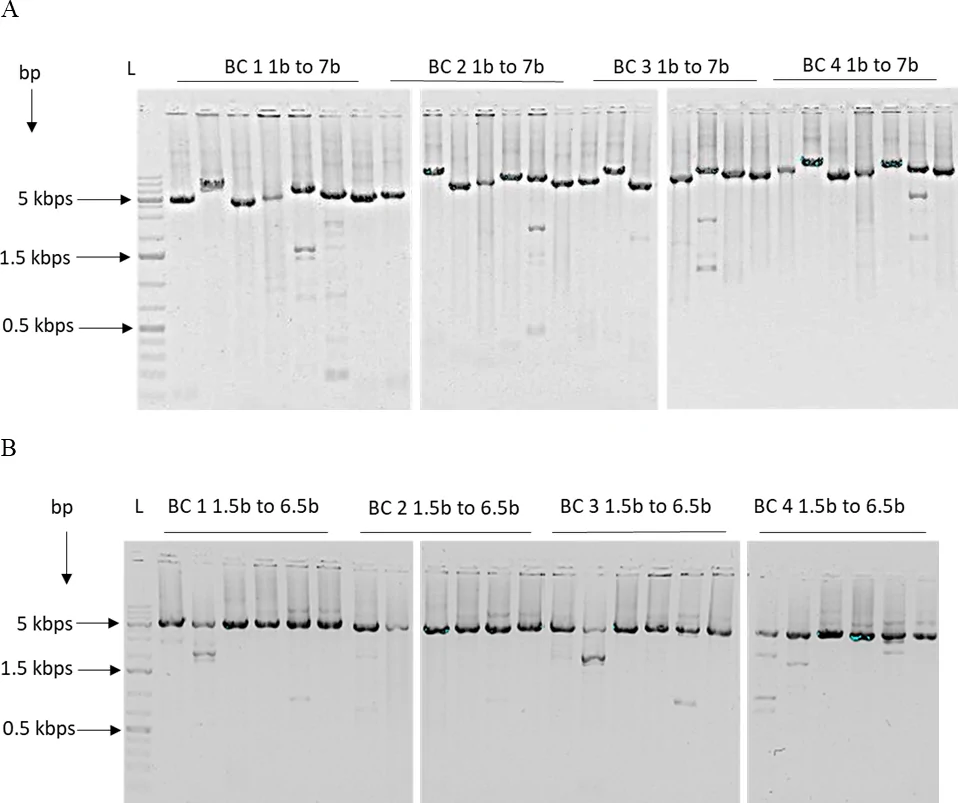
Agarose gel electrophoresis (1% agarose) of nested PCR products with 13 sets of primers across four samples, representing amplicon sizes ranging from ~4,000 to 6,400 base pairs. From left to right: bp, length of amplicon; L, DNA ladder; (**A**) PCR product by primer set 1b to 7b from sample BC 1 to BC 4; (**B**) PCR product by primer set 1.5b to 6.5b from sample BC 1 to BC 4. BC# refers to sample IDs as listed in Table 1.

All positive samples were subjected to nested PCR amplification using 13 overlapping primer sets (Table S1) to produce tiled amplicons that cover the entire ~35 kb human adenovirus F41 genome. Gel electrophoresis confirmed the expected amplicon sizes (~4,000–6,500 base pairs) with strong, distinct bands. An example of four samples is depicted in Fig. 2, and the gel electrophoresis results of the rest of the samples are represented in Fig. S1. The amplicons generated from the same sample were combined into a single pool for downstream processing, including purification and sequencing.

The pooled and purified amplicons from each sample were sequenced using the Oxford Nanopore Technologies platform. A total of 1,276,531 classified reads across all the samples were generated (Table S3). The produced sequencing reads were filtered to a size range of 4,100–6,000 base pairs, and the number of filtered reads across all wastewater samples ranged from 24,115 to 82,440 (Table S3). A positive control sample (BC 15 tak reference) of HAdV-F41 was sequenced with the wastewater samples, from which 70,000 reads were recovered after sequencing (Table S3).

**Fig 3:**
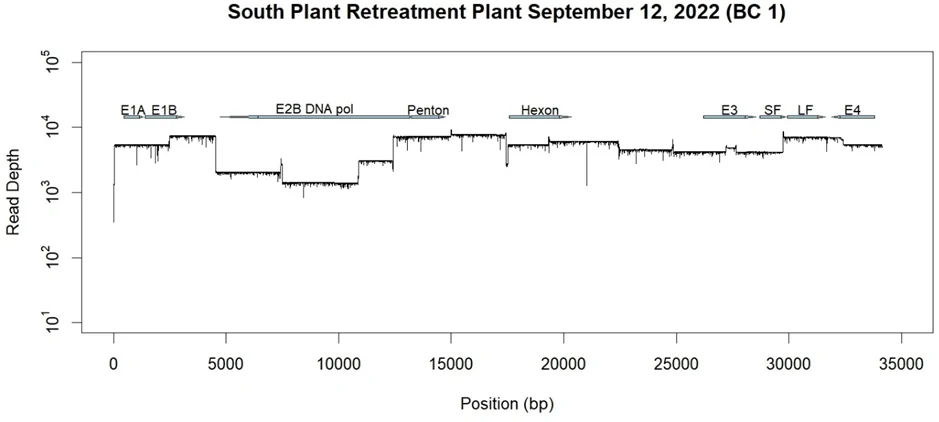
Demonstration of sequence read depths of sample BC 1 at each nucleotide position across the assembly in log scale, measured with Samtools depth (version 1.15.1), and the graph was created using RStudio. Here, the mean read depth is 4,966.16 (3.7 on a log scale). The genes, including E1A, E1B, E2B DNA pol, penton, hexon, E3, SF, LF, and E4, are annotated in the graph according to gene regions, and the direction of transcription for each gene is represented by the direction of arrowheads.

After *de novo* assembly with Flye, either single contigs spanning the whole genome or multiple contigs collectively covering the full genome were generated. For fragmented assemblies, contigs were merged based on overlaps or, when gaps remained, ordered by BLAST search, with gaps resolved using Medaka polishing. The consensus was mapped to closely related sequences obtained from a BLAST search at NCBI (Table 1). We successfully sequenced and assembled the genome of HAdV-F41 from wastewater, ranging in length from 29,004 to 34,165 bases (Table 1). According to the nucleotide BLAST search, all sequences exhibited high sequence similarity (identity ranging from 99.84 to 99.99%) with complete-genome sequences in NCBI GenBank, which are circulating in Japan, the United Kingdom, Germany, Spain, and Iraq (Table 1). The genome assemblies from this study comprised complete or partial genomes (>84%) containing open reading frame (ORF) regions, as indicated by their sequence lengths relative to similar BLAST reference genomes (Table 1). Most of the sequences (>99%) were recovered, including both structural and non-structural genes, such as E1A, E1B, E2, E3, E4, penton, hexon, SF, and LF (Fig. 3; Fig. S2). In comparison, two of the samples showed incomplete recovery (84.89% and 97.42%) at certain ORF sites, specifically at the 3′ and 5′ ends of the genome. Sample BC 12 lacked the inclusion of E1A, E1B 19K, partial E1B 55K, a portion of the LF gene, and E4 (Fig. S2). Sample BC 14 failed to recover the partial region of E4, including E4 ORF2 and E4 ORF3 (Fig. S2). Across all size-selected reads (4,100–6,000 bp), on average, 99.36% of reads were mapped to the BLAST accessions (Table 1). The mean coverage depths of reads to the genome assemblies ranged from 3.31 × 10^3^ to 1.17 × 10^4^ reads (Table 1). An example of the coverage depth of reads at each site for genomes and gene annotations is demonstrated in Fig. 3. The coverage depth of reads with annotations for the other samples is represented in Fig. S2.

**Fig 4:**
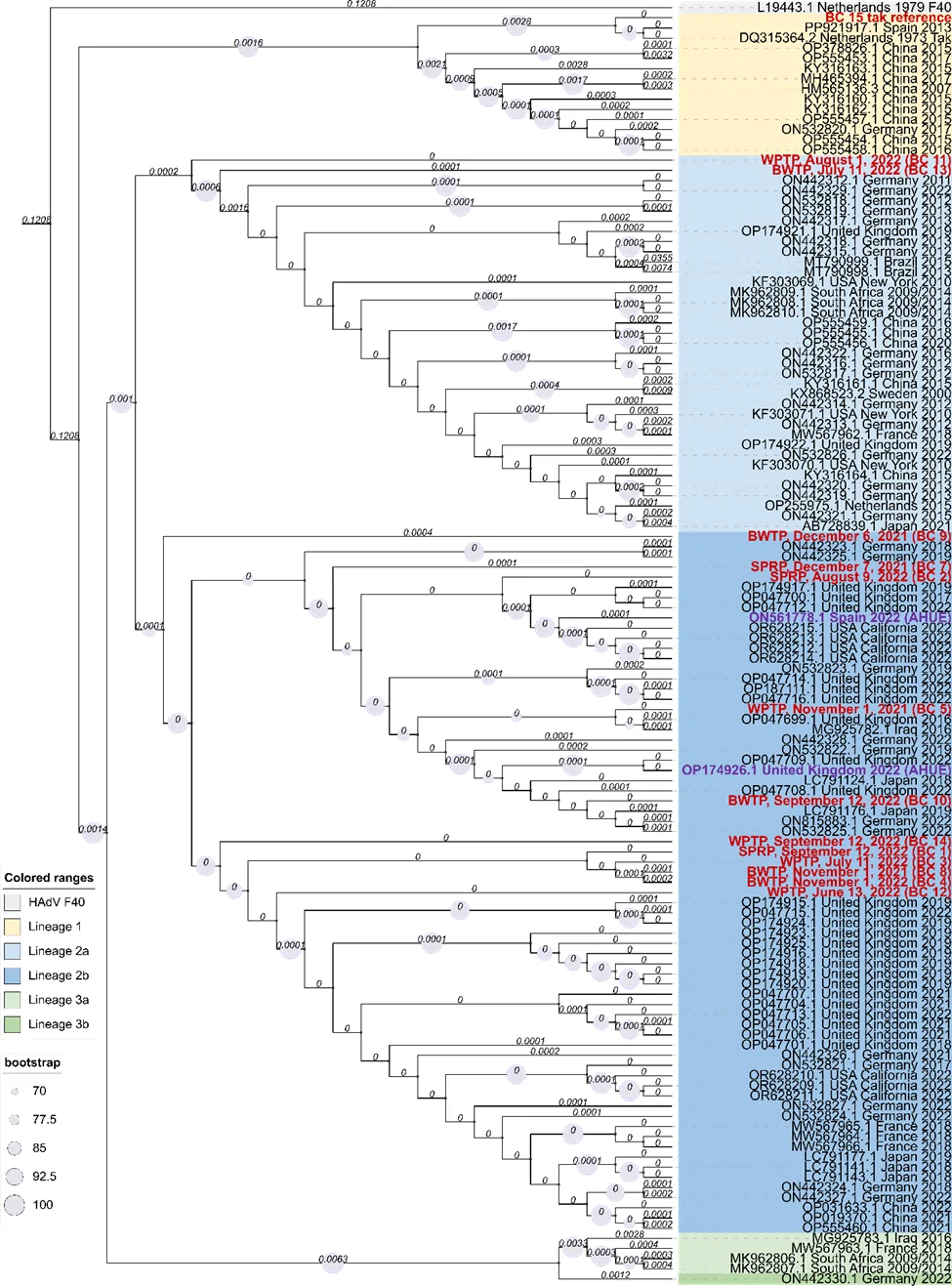
The phylogenetic tree obtained from the alignment of whole-genome sequences of HAdV-F41 from this study (red font) with 111 HAdV-F41 accessions from NCBI GenBank and HAdV-F40 accession as outgroup. Each cluster of different lineages is represented with a unique color. AHUE-associated strains are highlighted in purple. The tree was constructed using the maximum-likelihood method with IQ-TREE (version 2.4.0) and 1,000 ultrafast bootstrap replicates. Bootstrap values greater than 70 are represented by circles in five categories, with circle size proportional to the bootstrap value. Branch lengths, indicating genomic divergence among sequences, are shown for each branch of the tree.

The consensus genome derived from each sample was aligned with 111 reference genomes from NCBI GenBank representing HAdV-F41 (Table S1) and a prototype of HAdV-F40 (accession L19443.1) (6). A maximum likelihood phylogenetic tree with 1,000 ultrafast bootstrap replicates was constructed (Fig. 4). The human adenovirus F40 genome was positioned as an outgroup following a previous study (Fig. 4) (6). As expected, the positive control (BC 15 tak reference) was genomically clustered with lineage 1, confirming the reliability of the sequencing approach (Fig. 4). However, due to truncated assemblies of BC 12 and BC 14, a portion of the genome was missing from the whole-genome alignment. Because the alignment retained all genomic regions present in other sequences and was not trimmed prior to analysis, these strains have a higher proportion of missing sites than other taxa.

The resulting tree shows that out of 13, 11 genomes (BC 1 to 10, BC 12, and BC 14) clustered in lineage 2b, along with 58 genome accessions from GenBank circulating in Germany, the United Kingdom, Spain, California (USA), Iraq, Japan, France, and China from 2016 to 2022, as defined in HAdV-F41 classification (Fig. 4) (6). Within lineage 2b, two AHUE-associated reference accessions, OP174926.1 (UK) and ON561778.1 (Spain), highlighted with purple font (26, 47), formed a closely related sub-cluster (branch length <0.001) with four sequences (BC 7, BC 2, BC 5, and BC 10), along with multiple GenBank sequences that circulated between 2016 and 2022 in various regions of the world (Fig. 4). Additional samples of the same sub-lineage (except for BC 9) were clustered together, indicating high sequence similarity (branch lengths <0.001) among the sequences, along with previously reported globally circulating GenBank accessions from 2017 to 2022 (Fig. 4). The sample BC 9 appeared as a separate branch with a branch length of 4.3e^−4^ within the lineage 2b cluster (Fig. 4). Several nodes in this cluster showed strong bootstrap support (>95%), while others had moderate to low support (70%–85%), limiting the resolution needed to fully determine the branching order (48). However, all branch lengths in this cluster were very short (<0.001) regardless of bootstrap values, indicating high sequence similarity among strains.

Two genomes (BC 11 and BC 13) circulating in 2022 belonged to lineage 2a, along with 35 accessions from GenBank found in Germany, the United Kingdom, Brazil, New York (USA), South Africa, China, Sweden, France, and the Netherlands from 2000 to 2022, according to the previously reported classification of HAdV-F41 (6) (Fig. 4). Both sequences were highly similar (branch lengths <0.001) and positioned as separate peripheral branches within the cluster of lineage 2a (Fig. 4). Further phylogenetic analysis of the hexon, penton base, SF, and LF genes is presented in Fig. S3 to S6, which demonstrate evidence of intra-lineage diversification and inter-lineage similarities (6). In all gene phylogenetic trees, the positive control (BC 15 tak reference) clustered with other lineage 1 strains from NCBI GenBank, as expected.

In the phylogenetic tree representing the hexon, penton base, and LF genes, most of the lineage 2b strains obtained from this study were clustered with the majority of lineage 2b sequences from NCBI GenBank, indicating consistency with the tree topology of the whole-genome sequences, with a few exceptions (Fig. S3 to S5). For the hexon gene, the lineage 2a strains from this study were grouped with those from lineage 2b, forming a distinct branch within the lineage 2b clade (Fig. S3). According to the penton base phylogeny, one lineage 2b strain (BC 9) clustered with lineage 2a strains from this study, as well as lineage 2a sequences from GenBank, along with one lineage 2b strain (accession OP174921.1) from GenBank; two lineage 1 strains from GenBank were also grouped with the same cluster (Fig. S4). Similarly, for the LF gene, most lineage 2b strains from this study formed a clade with other lineage 2b sequences; however, a few strains of lineage 2a from GenBank, along with one lineage 2a strain (BC 11) from this study, clustered with most of the strains of lineage 2b. In contrast, another lineage 2a strain from this study (BC 13) grouped with the lineage 2a clade in Fig. S5. The clustering patterns suggest the possibility of shared genetic similarities between lineage 2a and 2b at all three gene sites, as well as shared genetic relatedness among lineages 1, 2a, and 2b for the penton base.

In contrast, the SF gene phylogeny did not align with the whole-genome clustering pattern, as shown in Fig. S6. All the lineage 2b and lineage 2a sequences from this study, along with most of the lineage 2b and lineage 2a sequences, and one lineage 1 strain from BLAST, formed a single cluster (Fig. S6). The rest of the lineage 1, 2a, and 2b accessions were also grouped, clustering as a sister clade to the previous cluster (Fig. S6).

The clustered HAdV-41 whole-genome sequences recovered directly from wastewater samples showed a limited number of genomic variants relative to the reference genome (in this case, the earliest-detected sequence in this cluster [BC 8 specifically]), with 5–10 mutations per sequence (Table 2). The majority of detected variants were single-nucleotide polymorphisms (SNPs), predominantly transition substitutions, including A→G, G→A, C→T, and T→C. Only one transversion substitution (C→A at genome position 6190) was identified among the analyzed sequences. In addition to SNPs, insertion and deletion events were observed, including a deletion at genome position 1777, a 5-bp deletion (AGGAC) at position 1889, and a cytosine insertion at position 17113. Several substitutions were shared among multiple wastewater-derived genomes, particularly among samples collected from the same treatment plant, suggesting the circulation of closely related HAdV-41 variants within the sampled wastewater systems. Sample B4 exhibited the highest mutational burden, containing 10 genomic variants relative to the reference sequence.

**TABLE 2:** Genomic substitutions and indels identified among clustered wastewater-derived HAdV-41 whole-genome sequences^*a*^

Sample		BC 8	BC 12	BC 3	BC 14	BC 1	BC 4
Site		BWTP	WPTP	WPTP	WPTP	SPTP	BWTP
Date		Nov-21	Jun-22	Jul-22	Sep-22	Sep-22	Nov-22
Genome position^*b*^	Variant types						
1039	del-T						
1335	T→C						
1777	C→T						
1889	del-AGGAC						
3047	A→G						
6190	C→A						
11278	G→A						
16162	G→A						
17113	ins-C						
20695	A→G						
23444	T→C						
24401	G→A						
29478	A→G						
33907	G→A						
34106	C→T						
Deletions			2	2	2	2	2
Insertions			1				
SNPs	Transition		2	4	4	5	7
	Transversion						1
							
Total variants			5	6	6	7	10

^*a*^ Shaded cells indicate the presence of a variant in the corresponding sequence. SNP, single nucleotide polymorphism; del, deletion; ins, insertion. Total mutations represent the number of variants identified per sequence relative to the reference genome (BC 8). Transition substitutions included A↔G and C↔T changes, while transversions included purine-pyrimidine substitutions.

^*b*^ Genome positions are based on the reference genome coordinates (BC 8).

**Fig 5:**
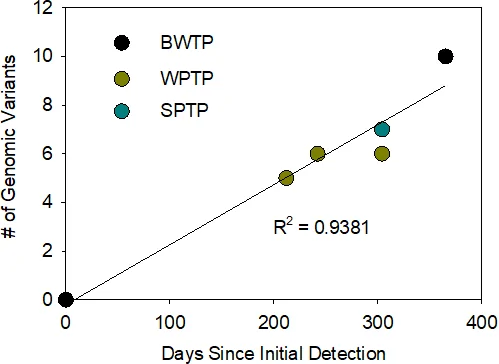
Temporal accumulation of genomic variants within a clustered lineage of wastewater-derived HAdV-41 genomes. The number of genomic variants was plotted against days since the initial detection of the clustered lineage in Seattle wastewater. Colors indicate wastewater treatment plant origin. The solid line represents the linear regression trend.

A cluster of closely related HAdV-41 genomes persisted in Seattle wastewater over time and accumulated a limited number of genomic variants relative to the earliest detected sequence (Fig. 5). The number of genomic variants increased progressively from 0 to 10 mutations over 406 days following the initial detection of the clustered lineage. Linear regression analysis estimated a temporal accumulation rate of approximately 0.0247 genomic variants per day, indicating limited genomic divergence within the clustered wastewater-derived HAdV-41 lineage over the sampling period.

Pairwise comparison between Seattle wastewater-derived HAdV-41 genomes and closely related AHUE-associated clinical isolates from England identified only a small number of nucleotide differences (Table 3). Comparison of BC 10 with isolate OP174926.1 revealed four SNPs, while BC 2 differed from isolate ON561778.1 by five SNPs across the genome. Most substitutions were transition mutations, with only one transversion detected at genome position 29800 (C→A). The limited genomic divergence between the wastewater-derived genomes and the AHUE-associated isolates suggests that genetically related HAdV-41 lineages were circulating in Seattle wastewater during the sampling period. Furthermore, the observed level of variation was comparable to that detected among temporally persistent wastewater-derived genomes, supporting the continued circulation of closely related HAdV-41 strains within the Seattle wastewater system.

**TABLE 3:** Pairwise genomic variants between Seattle wastewater-derived HAdV-41 genomes and closely related AHUE-associated clinical isolates from England^*a*^

Comparison	Position	Variant
OP174926.1 vs BC 10	4316	A→G
OP174926.1 vs BC 10	5094	T→C
OP174926.1 vs BC 10	11662	T→C
OP174926.1 vs BC 10	23246	A→G
ON561778.1 vs BC 2	734	A→G
ON561778.1 vs BC 2	1627	T→C
ON561778.1 vs BC 2	12085	A→G
ON561778.1 vs BC 2	22918	T→C
ON561778.1 vs BC 2	29800	C→A

^*a*^ Genome positions are based on the reference genome coordinates. Variants represent nucleotide differences between wastewater-derived HAdV-41 genomes and the closest related clinical whole-genome sequences from AHUE patient isolates.

## DISCUSSION

In this study, we demonstrate that whole-genome sequencing of HAdV-F41 is a successful approach for wastewater-based epidemiology, enabling the characterization of circulating lineages in a retrospective setting and contributing to the production of robust surveillance data. The study revealed the recovery of complete or partial HAdV-F41 genomes from 13 wastewater samples collected in Seattle, USA, between November and December 2021 and June to September 2022, with viral loads ranging from 1,210.5 to 8,394 GC/L. We successfully applied overlapping PCR-amplicon-based sequencing on the Oxford Nanopore platform, achieving our goal of obtaining whole-genome sequences of HAdV-F41 from wastewater samples.

Pathogen surveillance is crucial for mitigating the spread of infections. Genomic surveillance enhances surveillance data by enabling strain-level detection to identify disease-causing lineages (49). The clinical genomic surveillance of pathogens produces data on an individual basis rather than providing an actual scenario of circulating and emerging pathogens at the community level (49–51). Wastewater-based genomic surveillance scales up this limitation by introducing a community-level sampling approach, which aids in detecting circulating strains from both symptomatic and asymptomatic individuals, as well as emerging strains (49, 51, 52). For specific viruses, qPCR or digital PCR provides a reliable and sensitive approach for detection; however, these methods are limited to detecting shorter targets of DNA or RNA, which may raise questions about the accuracy of genotyping determinations (49, 51, 53). Whole-genome sequencing approaches overcome this limitation by providing comprehensive and accurate genotyping (52), thereby supporting ongoing public health investigations and offering insights into potential associations with significant disease outbreaks. In this study, the lineage of circulating HAdV-F41 is investigated, contributing to wastewater-based surveillance data by providing insights into community-level circulation of HAdV-F41, presumably from both symptomatic and asymptomatic individuals.

Methods currently used for wastewater-based viral surveillance have primarily relied on commercially available PCR primers targeting specific gene regions, rather than whole genomes, which restricts their resolution for lineage- or strain-level monitoring of certain viruses (2). For example, most of the PCR primers used for HAdV-F are incompetent to differentiate between HAdV-F40 and F41 reliably (2). Growing evidence on the whole-genome sequencing of viruses from wastewater, such as SARS-CoV-2 (52, 54), hepatitis E (55), norovirus GII (56, 57), influenza A virus (58, 59), and respiratory syncytial virus A (58), indicates that wastewater surveillance with whole-genome recovery enhances disease surveillance and provides public health insights.

There is evidence of cell culture (60), bait-based enrichment (61), and metagenomic sequencing (62) approaches to yield the whole genome of HAdV using clinical samples, each offering procedure-specific advantages and limitations. Cell culture is a time-intensive technique that can only be used to detect data from infectious particles and cellular genomic data, which may interfere with sequencing reads (63). The bait enrichment process may fail to recover high-throughput sequencing data at low viral loads (64). Advancements in sequencing techniques, particularly the introduction of multiplexing and the minimization of off-target genomic data, have the potential to reduce costs and increase sequencing throughput, as demonstrated for SARS-CoV-2 (65), hepatitis C virus (66, 67), and Zika virus (68). Amplicon-based sequencing offers a cost-effective, high-throughput, and flexible approach for generating whole-genome sequencing data for HAdV-F species (2). This aproach has been used for the successful recovery of whole-genome sequences using overlapping-amplicon-based methods from RNA viruses, including Ebola (filovirus), respiratory syncytial virus (*Pneumoviridae*), Zika (flaviviruses), dengue (flaviviruses), Usutu virus (flaviviruses), and SARS-CoV-2 (coronavirus) (65, 69–72). Multiple studies have reported whole-genome sequencing of SARS-CoV-2 from wastewater samples using a pooled amplicon-based method (e.g., ARTIC), which enables broader surveillance than short amplicon RT-PCR assays (52, 73–76). During the AHUE surge in the UK, a similar approach was successfully deployed, in which researchers recovered DNA virus-like HAdV-F sequences from HAdV-positive stool samples from pediatric cases (2). In this study, this method was applied to recover HAdV-F41 sequences from archived wastewater samples collected during a period when AHUE cases were increasing. The findings of this study highlight the reliability of the tiled PCR amplicon-based approach for generating whole-genome sequences from wastewater, enabling the tracking of clinically relevant viral lineages within the community.

In this study, complete or partial HAdV-F41 genomes covering ORF regions containing both structural and non-structural protein transcription sites were recovered. A previous study reported decreased coverage within the L3 hexon and L4 hexon assembly protein regions in clinical samples (2). In our research, this limitation was overcome through the design of alternative primers and a nested PCR approach, which enabled the recovery of both structural and non-structural protein-coding gene regions, such as E1A, E1B, E2, E3, E4, penton, hexon, and fiber, in most samples (Fig. 3; Fig. S2). The nested PCR approach increased the coverage depth (mean coverage depth ranged between 10^3^ and 10^5^ reads) (Table 1) (Fig. 3; Fig. S2), contributing to robust genome assemblies and preventing amplicon dropouts in internal regions of the genomes. Despite these improvements, two samples showed incomplete recovery. We believe this could have been caused by DNA fragmentation during purification or inhibition, but more work needs to be done to resolve these issues. For sample BC 14, part of the ORF genomic region was missing, specifically E4 ORF2 and E4 ORF3 (Fig. S2). Sample BC 12 failed to recover multiple gene regions, including E1A, E1B 19K, partial E1B 55K, a portion of the long fiber gene, and E4 (Fig. S2). The early genes, including E1A, E1B, E2, E3, and E4, encode proteins that are essential for activating transcription, assisting viral replication, and manipulating the host cell environment to encourage viral propagation (77–81). So, the absence of these early gene regions in the two samples highlighted a limitation of this study, as incomplete coverage failed to provide a comprehensive view of these regions of the viral genome. Moreover, it restricts the detection of mutations, including insertions, deletions, and single-nucleotide polymorphisms, in early non-structural protein-coding regions, limiting the characterization of viral regulatory and replication functions (82, 83). The absence of part of the long fiber gene also limits the detection of mutations and characterization of the structural fiber protein, which plays a crucial role in the infection cycle by recognizing the primary receptor (84). Moreover, missing positions are treated as non-informative sites in maximum likelihood analyses, resulting in reduced phylogenetic resolution and less confident placement of BC 12 and BC 14 in the whole-genome phylogenetic tree (Fig. 4), as well as BC 12 in the long fiber gene phylogeny (Fig. S5).

Phylogenetic analysis of whole-genome sequences, incorporating wastewater samples and previously reported AdV-F41 and AdV-F40 sequences from NCBI GenBank, revealed that the most dominant genotype clustered within Lineage 2b, representing 84.6% (11/13) of the samples (Fig. 4). Lineage 2a, representing 15.4% (2/13), was the second most prevalent circulating strain (Fig. 4). No sequence clustered with lineages 1 and 3. A previous study demonstrated that lineage 2, comprising two sub-lineages (2a and 2b), incorporated the majority of Adv-F41 sequences collected between 2000 and 2022 from multiple regions, including Germany, Belgium, Japan, the USA, Sweden, China, South Africa, France, the UK, and Iraq (6). In our analysis, we further identified that lineage 2 was also circulating in Spain and the Netherlands during this period (Fig. 4). The data from this study suggest that two sub-lineages (2a and 2b) of lineage 2 HAdV-F41 were circulating in Seattle, Washington, between 2021 and 2022. Between June 2021 and May 2022, five cases of acute gastroenteritis in children testing positive for HAdV-F41 were reported in the same region; however, it remains unclear whether the lineages found from this study were associated with these cases, as no sequencing was performed to determine the relevant strains (85).

Phylogenetic analysis at the gene level, based on nucleotide sequencing, revealed a similar phylogenetic grouping for the hexon, penton, SF, and LF genes, as previously described (6) (Fig. S3b). The hexon gene, responsible for encoding the major capsid protein, contains hypervariable regions that follow the clustering pattern of whole-genome phylogeny as described before (6). The lineage 2b from our study followed a similar pattern, while lineage 2a showed some genetic similarities with lineage 2b, suggesting relatedness between the two lineages. The penton (highly conserved region) and the LF gene phylogeny also highlighted lineage structure through clustering patterns but revealed some deviations that reflect shared genomic similarities among lineages. The SF gene region was highly conserved, reflecting genetic similarities among strains of lineages 1, 2a, and 2b, and exhibiting limited evolution, resulting in low phylogenetic resolution for distinguishing between lineages. These results demonstrated that mechanisms of evolutionary pressure affect genes individually. Similar results have been observed in adenovirus D, where certain genes, such as E3, penton, and hexon, evolve more rapidly (86). In adenovirus D, evidence of gene recombination has been observed among various strains (87).

Since March 2022, cases of acute hepatitis linked to HAdV-F41 have been reported in the United Kingdom, subsequently appearing in multiple geographic locations (27, 28). From October 2021 to May 2022, similar cases were reported in regions including the USA, Europe, Asia, and Central America (28). Two accessions from NCBI GenBank, OP174926.1 and ON561778.1, from the United Kingdom and Spain, respectively, have been confirmed as belonging to AHUE (26, 47). Both sequences are from lineage 2b, and, notably, 11 of 13 sequences in this study cluster within the same lineage in the whole-genome phylogeny (Fig. 4). Among these, four sequences form a close cluster, showing higher similarity to AHUE-associated sequences. Therefore, lineage-specific clustering and proximity to clinically relevant sequences among intra-lineage sequences may suggest molecular evidence of an ancestral connection across different regions. The findings would be further supported if current clinical evidence from the same region were linked to wastewater data, as has been shown for SARS-CoV-2 (76). In the USA, a case series in Alabama between 1 October 2021 and 28 February 2022 showed that AHUE was associated with at least three different HAdV-F41 variants based on the hypervariable regions of the hexon gene (24). A total of 100 patients with AHUE, attributed to HAdV-F41 infections, were diagnosed in the USA. Variants were identified using a similar method that targeted the hypervariable regions of the hexon gene (88). However, these studies did not perform whole-genome genotyping to assign strains to previously known lineages (6), highlighting a gap in understanding their relevance to circulating strains worldwide. Overall, while the direct clinical link to our findings remains unclear, the whole-genome sequencing performed with the tiled amplicon method in this study provides molecular evidence of public health significance by illustrating phylogenetic connections between circulating strains and previously reported clinically meaningful HAdV-F41 serotypes from other regions. These findings also lay the groundwork for future research into mutations within the HAdV-F41 genome, which may provide deeper insights into the patterns of genetic evolution and disease outbreaks (23).

One limitation of this study is that we were unable to recover the haplotype or identify the co-circulating variants of HAdV-F41 from the same sample, as these may not be captured by consensus sequences generated from *de novo* assemblies. This means that if two different viruses are found in the samples at different concentrations, we may not be able to assemble sequences for both viruses, but only for the dominant strain. We used the medaka variant pipeline, but only variants with a single base substitution were identified (data not shown). On the other hand, there is evidence of haplotyping for detecting co-circulating variants of SARS-CoV-2, poliovirus, and coxsackievirus from wastewater samples using both short- and long-read data (89, 90). However, further study is necessary to design a haplotyping approach for HAdV-F41 strains. Another limitation is the possibility that the regions at both ends (3′ and 5′ untranslated regions [UTR]) of the HAdV-F41 genome are not fully sequenced. In HAdV, the 5′ UTR facilitates late gene expression by enabling cap-independent initiation and contributing to mRNA localization and stability (91). Therefore, the protocol design needs further modification to capture these end regions.

The detection of a closely related cluster of HAdV-41 genomes over an extended sampling period suggests a sustained circulation of a stable viral lineage within the Seattle wastewater system. Although genomic variants accumulated progressively over time, the overall level of divergence remained low, with only 5–10 variants identified across approximately 406 days of sampling. This limited temporal divergence is consistent with the relatively stable double-stranded DNA genome of adenoviruses (91), which generally evolve more slowly than RNA viruses due to higher replication fidelity. The estimated accumulation rate of approximately 0.0247 genomic variants per day further supports the persistence of a genetically conserved HAdV-41 lineage within the sampled population. Additionally, the close genetic relationship between the wastewater-derived genomes and AHUE-associated clinical isolates from England indicates that highly similar HAdV-41 strains were circulating internationally during the study period. Together, these findings demonstrate the utility of wastewater genomic surveillance for tracking the persistence and temporal dynamics of clinically relevant HAdV-41 lineages in the community.

This study highlighted the implications and feasibility of a tiled amplicon-based whole-genome sequencing approach to recover circulating human adenovirus F41 lineages from the complex wastewater matrix. It provided insights into the importance of whole-genome sequencing for public health surveillance. Despite this study being based on retrospective samples, applying this approach in a contemporary setting will be beneficial for monitoring ongoing circulation and potential outbreak dynamics. The primers from this study can be reused for future research, as they were specifically designed to target HAdV-F41 and produced high-quality, high-volume DNA amplicons, serving as a basis for generating high-throughput sequencing reads. Moreover, this methodological framework will serve as a foundation for future applications in conducting whole-genome sequencing research for wastewater-based epidemiology, targeting other adenovirus types.

## Data Availability

The genome sequences generated in this study have been deposited in GenBank under accession numbers PZ360987–PZ360999.

## SUPPLEMENTAL MATERIAL

Supplemental Material spectrum.00471-26-s0001.docx Tables S1 to S3; Fig. S1 to S6.

ASM does not own the copyrights to Supplemental Material that may be linked to, or accessed through, an article. The authors have granted ASM a non-exclusive, world-wide license to publish the Supplemental Material files. Please contact the corresponding author directly for reuse.
