# Supplementary material for: Whole-genome sequencing of adenovirus 41 directly from wastewater using nested overlapping PCR and MinION
Source: Microbiol Spectr. 2026 Aug 5;14(9):e00471-26. doi: 10.1128/spectrum.00471-26 (PMC13532293; doi:10.1128/spectrum.00471-26)
Supplement: Supplementary file 1 [file spectrum.00471-26-s0001.docx]

**Whole-genome sequencing of Adenovirus 41 directly from wastewater using nested overlapping PCR and MinION**

Supplementary Materials:

**Supplementary Table 1:** List of accessions, location of accessions and year of Human Adenovirus F41 from NCBI GenBank; Accessions attributed to hepatitis of unknown etiology (AHUC) are highlighted with red font

DQ315364.2_Netherlands_1973_Tak

HM565136.3_China_2007

OP555460.1_China_2021

OP555459.1_China_2016

OP555458.1_China_2016

OP555457.1_China_2015

OP555456.1_China_2020

OP555455.1_China_2018

OP555454.1_China_2015

OP555453.1_China_2017

OP378826.1_China_2015

MW567966.1_France_2018

MW567965.1_France_2018

MW567964.1_France_2018

MW567963.1_France_2018

MW567962.1_France_2018

MH465394.1_China_2017

KX868523.2_Sweden_2000

MG925783.1_Iraq_2016

MG925782.1_Iraq_2016

OR628213.1_USA(California)_2022

PP921917.1_Spain_2013

OR628215.1_USA(California)_2022

OR628214.1_USA(California)_2022

OR628212.1_USA(California)_2022

OR628211.1_USA(California)_2022

OR628210.1_USA(California)_2022

OR628209.1_USA(California)_2022

OP255975.1_Netherlands_2015

OP174926.1_United_Kingdom_2022

OP174925.1_United_Kingdom_2019

OP174924.1_United_Kingdom_2019

OP174923.1_United_Kingdom_2019

OP174922.1_United_Kingdom_2019

OP174921.1_United_Kingdom_2019

OP174920.1_United_Kingdom_2019

OP174919.1_United_Kingdom_2019

OP174918.1_United_Kingdom_2019

OP174917.1_United_Kingdom_2019

OP174916.1_United_Kingdom_2019

OP174915.1_United_Kingdom_2019

OP047716.1_United_Kingdom_2022

OP047715.1_United_Kingdom_2022

OP047714.1_United_Kingdom_2022

OP047713.1_United_Kingdom_2022

OP047712.1_United_Kingdom_2022

OP047709.1_United_Kingdom_2022

OP047708.1_United_Kingdom_2022

OP047707.1_United_Kingdom_2021

OP047706.1_United_Kingdom_2021

OP047705.1_United_Kingdom_2021

OP047704.1_United_Kingdom_2021

OP047701.1_United_Kingdom_2018

OP047700.1_United_Kingdom_2017

OP047699.1_United_Kingdom_2016

OP187111.1_United_Kingdom_2022

ON532827.1_Germany_2022

ON532826.1_Germany_2022

ON532825.1_Germany_2022

ON532824.1_Germany_2022

ON532823.1_Germany_2019

ON532822.1_Germany_2019

ON532821.1_Germany_2017

ON532820.1_Germany_2017

ON532819.1_Germany_2013

ON532818.1_Germany_2012

ON532817.1_Germany_2012

ON442330.1_Germany_2022

ON442329.1_Germany_2022

ON442328.1_Germany_2022

ON442327.1_Germany_2022

ON442326.1_Germany_2021

ON442325.1_Germany_2019

ON442324.1_Germany_2018

ON442323.1_Germany_2018

AB728839.1_Japan_2021

KF303069.1_USA(New_York) _2010

KF303070.1_USA(New_York)_2010

KF303071.1_USA(New_York)_2010

KY316160.1_China_2015

KY316161.1_China_2015

KY316162.1_China_2015

KY316163.1_China_2015

OP031633.1_China_2022

KY316164.1_China_2015

MT790998.1_Brazil_2013

MT790999.1_Brazil_2015

ON561778.1_Spain_2022

ON442312.1_Germany_2011

ON442313.1_Germany_2012

ON442314.1_Germany_2012

ON442315.1_Germany_2012

ON442316.1_Germany_2012

ON442317.1_Germany_2013

ON442318.1_Germany_2013

ON442319.1_Germany_2013

ON442320.1_Germany_2013

ON442321.1_Germany_2015

ON442322.1_Germany_2016

MK962810.1_South_Africa_2009/2014

MK962809.1_South_Africa_2009/2014

MK962808.1_South_Africa_2009/2014

MK962807.1_South_Africa_2009/2014

MK962806.1_South_Africa_2009/2014

LC791177.1_Japan_2019

LC791176.1_Japan_2019

LC791143.1_Japan_2018

LC791141.1_Japan_2018

LC791124.1_Japan_2018

ON815883.1_Germany_2022

OP019370.1_China_2021

**Supplementary Table 2:** List of PCR primers of Human Adenovirus F41 used for amplicon production. The primer sets, 1, 2, 3, 4, 5, 6 & 7, correspond to the primary sets of amplicons. The primer sets 1.5, 2.5, 3.5, 4.5, 5.5 & 6.5 correspond to amplicons that cover regions not amplified by the primary sets and overlap adjacent amplicons to provide complete coverage. “a” represents primers used for first-step PCR products of nested PCR amplification, “b” represents primers used for nested PCR products.

| Amplicon | Primer | Reference | Start Coordinate | End Coordinate | Type | Sequence |
| --- | --- | --- | --- | --- | --- | --- |
| 1a | Outer Adv-F41 0AF | DQ315364.2 | 14 | 37 | Forward | ATA CCT TAA AGC TGG AAA CGA GCC |
| 1a | Outer Adv-F41 5AR | DQ315364.2 | 4556 | 4539 | Reverse | CTG CGG GAA GAC GGC AGC |
| 1b | Nested Adv-F41 0BF | DQ315364.2 | 41 | 66 | Forward | ATG ATA ATG AGT GAG GAG GGG CTA GG |
| 1b | Nested Adv-F41 5BR | DQ315364.2 | 4572 | 4553 | Reverse | CGA AGT TAC CCC ACT CCT GC |
| 1.5a | Outer Adv-F41 2.5AF | DQ315364.2 | 2473 | 2490 | Forward | GCA GCG GTG TTT GCG TGG |
| 1.5a | Outer Adv-F41 7.5AR | DQ315364.2 | 7463 | 7444 | Reverse | GCA ACT GGT CCC GTT TAT GC |
| 1.5b | Nested Adv-F41 2.5BF | DQ315364.2 | 2504 | 2525 | Forward | GGT GTC TGC CAG AGG TTG TAC G |
| 1.5 b | Nested Adv-F41 7.5 BR | DQ315364.2 | 7514 | 7490 | Reverse | CTT CGT CAC CTA CGA CGT TGA GAC C |
| 2a | Outer Adv-F41 5AF | DQ315364.2 | 4539 | 4556 | Forward | GCT GCC GTC TTC CCG CAG |
| 2a | Outer Adv-F41 10 AR | DQ315364.2 | 10904 | 10876 | Reverse | GGC TCA GCA ATG TTA AGC ATA GCT TCT CG |
| 2b | Nested Adv-F41 5BF | DQ315364.2 | 4553 | 4572 | Forward | GCA GGA GTG GGG TAA CTT CG |
| 2b | Nested Adv-F41 10BR | DQ315364.2 | 10939 | 10912 | Reverse | GGA TGT TGA CTA GGT CTA AAA GCC AGC G |
| 2.5 a | Outer Adv-F41 7.5 AF | DQ315364.2 | 7444 | 7463 | Forward | GCA TAA ACG GGA CCAG TTG C |
| 2.5 a | Outer Adv-F41 12.5 AR | DQ315364.2 | 12401 | 12377 | Reverse | CAA GAT ACG CTC TTC CTC GGT ACT C |
| 2.5 b | Nested Adv-F41 7.5 BF | DQ315364.2 | 7490 | 7514 | Forward | GGT CTC AAC GTC GTA GGT GAC GAA G |
| 2.5 b | Nested Adv-F41 12.5 BR | DQ315364.2 | 12473 | 12453 | Reverse | CAT ATC CAG AGC CGA GGA TGG |
| 3a | Outer Adv-F41 10 AF | DQ315364.2 | 10876 | 10904 | Forward | CGA GAA GCT ATG CTT AAC ATT GCT GAG CC |
| 3a | Outer Adv-F41 15 AR | DQ315364.2 | 15028 | 15007 | Reverse | GCT ACC ACG CTG TCA ATC ACG G |
| 3b | Nested Adv-F41 10 BF | DQ315364.2 | 10912 | 10939 | Forward | CGC TGG CTT TTA GAC CTA GTC AAC ATC C |
| 3b | Nested Adv-F41 15 BR | DQ315364.2 | 15045 | 15027 | Reverse | CAT AGC GCC TGG CAT CTG C |
| 3.5 a | Outer Adv-F41 12.5 AF | DQ315364.2 | 12377 | 12401 | Forward | GAG TAC CGA GGA AGA GCG TAT CTT G |
| 3.5 a | Outer Adv-F41 17.5 AR | DQ315364.2 | 17459 | 17436 | Reverse | CAA TGC TGT TCA AAG TGC TCT GCC |
| 3.5 b | Nested Adv-F41 12.5 BF | DQ315364.2 | 12453 | 12473 | Forward | CCA TCC TCG GCT CTG GAT ATG |
| 3.5 b | Nested Adv-F41 17.5 BR | DQ315364.2 | 17557 | 17538 | Reverse | GTC TGG CGT AAC ACA GGA GG |
| 4a | Outer Adv-F41 15 AF | DQ315364.2 | 15007 | 15028 | Forward | CCG TGA TTG ACA GCG TGG TAG C |
| 4a | Outer Adv-F41 20 AR | DQ315364.2 | 19367 | 19340 | Reverse | GGC GTA CAG ATT AAT GCT ATC GAA CCT G |
| 4b | Nested Adv-F41 15 BF | DQ315364.2 | 15027 | 15045 | Forward | GCA GAT GCC AGG CGC TAT G |
| 4b | Nested Adv-F41 20 BR | DQ315364.2 | 19399 | 19379 | Reverse | GAG GCG GTG TTG TGA GCC ATG |
| 4.5 a | Outer Adv-F41 17.5 AF | DQ315364.2 | 17436 | 17459 | Forward | GGC AGA GCA CTT TGA ACA GCA TTG |
| 4.5 a | Outer Adv-F41 22.5 AR | DQ315364.2 | 22447 | 22430 | Reverse | CTA CCT CCA GGA GAC GAG |
| 4.5 b | Nested Adv-F41 17.5 BF | DQ315364.2 | 17538 | 17557 | Forward | CCT CCT GTG TTA CGC CAG AC |
| 4.5 b | Nested Adv-F41 22.5 BR | DQ315364.2 | 22498 | 22477 | Reverse | CTA GGA GTA AAG CAT GGC TGG C |
| 5a | Outer Adv-F41 20 AF | DQ315364.2 | 19340 | 19367 | Forward | CAG GTT CGA TAG CAT TAA TCT GTA CGC C |
| 5a | Outer Adv-F41 25 AR | DQ315364.2 | 24853 | 24834 | Reverse | GGT GTC CCA TCT ACG GCT GG |
| 5b | Nested Adv-F41 20 BF | DQ315364.2 | 19379 | 19399 | Forward | CAT GGC TCA CAA CAC CGC CTC |
| 5b | Nested Adv-F41 25 BR | DQ315364.2 | 24875 | 24853 | Reverse | GTG GAG ATC CTG CGA TGG AAA CG |
| 5.5 a | Outer Adv-F41 22.5 AF | DQ315364.2 | 22430 | 22447 | Forward | CTC GTC TCC TGG AGG TAG |
| 5.5 a | Outer Adv-F41 27.5 AR | DQ315364.2 | 27252 | 27231 | Reverse | GCT GGG TAT TTG GAG TTA GTG C |
| 5.5 b | Nested Adv-F41 22.5 BF | DQ315364.2 | 22477 | 22498 | Forward | GCC AGC CAT GCT TTA CTC CTA G |
| 5.5 b | Nested Adv-F41 27.5 BR | DQ315364.2 | 27676 | 27655 | Reverse | CGT ATC AAG GTG GGA GAC TGC G |
| 6a | Outer Adv-F41 25 AF | DQ315364.2 | 24834 | 24853 | Forward | CCA GCC GTA GAT GGG ACA C C |
| 6a | Outer Adv-F41 30 AR | DQ315364.2 | 29768 | 29744 | Reverse | GCG AGA TTT GGA TGA GCA TGT TGC C |
| 6b | Nested Adv-F41 25 BF | DQ315364.2 | 24853 | 24875 | Forward | CGT TTC CAT CGC AGG ATC TCC AC |
| 6b | Nested Adv-F41 30 BR | DQ315364.2 | 29791 | 29767 | Reverse | GAC GAC ACT GAA GGT GAT GTT AGG C |
| 6.5 a | Outer Adv-F41 27.5 AF | DQ315364.2 | 27231 | 27252 | Forward | GCA CTA ACT CCA AAT ACC CAG C |
| 6.5 a | Outer Adv-F41 32.5 AR | DQ315364.2 | 32330 | 32303 | Reverse | CTT GGT ATC GAG AAA TTG TAA ACT GTG GC |
| 6.5 b | Nested Adv-F41 27.5 BF | DQ315364.2 | 27655 | 27676 | Forward | CGC AGT CTC CCA CCT TGA TAC G |
| 6.5 b | Nested Adv-F41 32.5 BR | DQ315364.2 | 32433 | 32405 | Reverse | CAC TGT AAT AAA CCT GGG TCT CTT CAA TG |
| 7a | Outer Adv-F41 30 AF | DQ315364.2 | 29744 | 29768 | Forward | GGC AAC ATG CTC ATC CAA ATC TCG C |
| 7a | Outer Adv-F41 35 AR | DQ315364.2 | 34157 | 34129 | Reverse | CGA GCC AAT ATG ATA ATG AGT GAG GAG GG |
| 7b | Nested Adv-F41 30 BF | DQ315364.2 | 29767 | 29791 | Forward | GCC TAA CAT CAC CTT CAG TGT CGT C |
| 7b | Nested Adv-F41 35 BR | DQ315364.2 | 34173 | 34153 | Reverse | ACC TTA AAG CTG GAA ACG AGC |

**Supplementary Table 3:** Overview of sequencing reads

| Sample ID | Passed total reads | Total read after filtering by size (%) | Number of filtered reads primarily mapped to BLAST HAdV F41 accessions **(as shown in Table 1 of the main text)** |
| --- | --- | --- | --- |
| bc 1 | 67253 | 36149 (53.75%) | 35977 (99.52%) |
| bc 2 | 101235 | 62287 (61.53%) | 62087 (99.68%) |
| bc 3 | 61836 | 36593 (59.18%) | 36439 (99.58%) |
| bc 4 | 107296 | 57277 (53.38%) | 56590 (98.80%) |
| bc 5 | 123287 | 82440 (66.87%) | 82098 (99.59%) |
| bc 7 | 89204 | 55675 (62.41%) | 54964 (98.72%) |
| bc 8 | 79786 | 49604 (62.17%) | 49114 (99.01) |
| bc 9 | 38464 | 24115 (62.69%) | 23460 (97.28%) |
| bc 10 | 108179 | 70386 (65.06%) | 70164 (99.68%) |
| bc 11 | 93251 | 61460 (65.91%) | 61449 (99.98%) |
| bc 12 | 54229 | 36688 (67.65%) | 36287 (98.91%) |
| bc 13 | 93010 | 60718 (65.28%) | 60346 (99.39%) |
| bc 14 | 109679 | 73823 (67.31%) | 73265 (99.24%) |
| bc 15 (tak reference) | 149822 | 70000 (46.72%) | 69993 (99.99%) |
| **Total =** | 1,276,531 | 777215 | 772,233 (99.36%) |

A

B

**Supplementary Figure 1**: Agarose gel electrophoresis **(**1% agarose) of nested PCR products with 13 sets of primers, representing amplicon sizes ranging from ~ 4000 to 5000 base pairs. From left to right: bp, length of amplicon; L, DNA ladder; (A) PCR product by primer set 1b to 7b ; (B) PCR product by primer set 1.5b to 6.5. BC# refers to the sample ID as listed in **Table 1**. Lanes were derived from separate gels processed under comparable experimental conditions and assembled into a composite image; splicing is indicated by gaps.

A

B

C

D

E

F

G

H

I

J

K

L

M

**Supplementary Figure 2 (A-M):** Demonstration of sequence read depths of sample bc 2 to 15 at each nucleotide position across the assembly in log scale, measured with Samtools depth (version 1.15.1) and graph created using RStudio. The genes, including E1A, E1B, E2B DNA pol, Penton, Hexon, E3, Short fiber (SF), Long fiber (LF), E4, are annotated in the graph according to gene regions, and the direction of transcription for each gene is represented with the direction of arrowheads.

**Supplementary Figure 3:** The phylogenetic tree of alignment of Hexon gene sequences of HAdV F41 from this study (red font) with 111 HAdV F41 accessions from NCBI GenBank and HAdV F40 accession as outgroup. Each cluster of different lineages is represented with a unique color. AHUC-associated strains are highlighted with purple font. The tree was constructed using the maximum likelihood method with IQ-TREE (version 2.4.0) and 1,000 ultrafast bootstraps. Bootstrap values greater than 70 are represented by circles in 5 categories, where the circle size is proportional to the bootstrap value. The branch lengths, indicating genomic divergence among sequences, are represented with scientific numbers along with each branch of the tree. The tree was annotated using iTOL v7.

**Supplementary Figure 4:** The phylogenetic tree of alignment of Penton gene of HAdV F41 from this study (red font) with 111 HAdV F41 accessions from NCBI GenBank and HAdV F40 accession as outgroup. Each cluster of different lineages is represented with a unique color. AHUC-associated strains are highlighted with purple font. The tree was constructed using the maximum likelihood method with IQ-TREE (version 2.4.0) and 1,000 ultrafast bootstraps. Bootstrap values greater than 70 are represented by circles in 5 categories, where the circle size is proportional to the bootstrap value. The branch lengths, indicating genomic divergence among sequences, are represented with scientific numbers along with each branch of the tree. The tree was annotated using iTOL v7.

**Supplementary Figure 5:** The phylogenetic tree of alignment of Long fiber gene of HAdV F41 from this study (red font) with 111 HAdV F41 accessions from NCBI GenBank and HAdV F40 accession as outgroup. Each cluster of different lineages is represented with a unique color. AHUC-associated strains are highlighted with purple font. The tree was constructed using the maximum likelihood method with IQ-TREE (version 2.4.0) and 1,000 ultrafast bootstraps. Bootstrap values greater than 70 are represented by circles in 5 categories, where the circle size is proportional to the bootstrap value. The branch lengths, indicating genomic divergence among sequences, are represented with scientific numbers along with each branch of the tree. The tree was annotated using iTOL v7.

**Supplementary Figure 6:** The phylogenetic tree of alignment of Short fiber gene of HAdV F41 from this study (red font) with 111 HAdV F41 accessions from NCBI GenBank and HAdV F40 accession as outgroup. Each cluster of different lineages is represented with a unique color. AHUC-associated strains are highlighted with purple font. The tree was constructed using the maximum likelihood method with IQ-TREE (version 2.4.0) and 1,000 ultrafast bootstraps. Bootstrap values greater than 70 are represented by circles in 5 categories, where the circle size is proportional to the bootstrap value. The branch lengths, indicating genomic divergence among sequences, are represented with scientific numbers along with each branch of the tree. The tree was annotated using iTOL v7.
